# The Unstable CCTG Repeat Responsible for Myotonic Dystrophy Type 2 Originates from an *Alu*Sx Element Insertion into an Early Primate Genome

**DOI:** 10.1371/journal.pone.0038379

**Published:** 2012-06-19

**Authors:** Tatsuaki Kurosaki, Shintaroh Ueda, Takafumi Ishida, Koji Abe, Kinji Ohno, Tohru Matsuura

**Affiliations:** 1 Division of Neurogenetics, Center for Neurological Diseases and Cancer, Nagoya University Graduate School of Medicine, Nagoya, Japan; 2 Department of Biological Sciences, Graduate School of Science, The University of Tokyo, Tokyo, Japan; 3 Department of Neurology, Okayama University Graduate School of Medicine, Dentistry and Pharmaceutical Sciences, Okayama, Japan; Louisiana State University, United States of America

## Abstract

Myotonic dystrophy type 2 (DM2) is a subtype of the myotonic dystrophies, caused by expansion of a tetranucleotide CCTG repeat in intron 1 of the zinc finger protein 9 (*ZNF9*) gene. The expansions are extremely unstable and variable, ranging from 75–11,000 CCTG repeats. This unprecedented repeat size and somatic heterogeneity make molecular diagnosis of DM2 difficult, and yield variable clinical phenotypes. To better understand the mutational origin and instability of the *ZNF9* CCTG repeat, we analyzed the repeat configuration and flanking regions in 26 primate species. The 3′-end of an *Alu*Sx element, flanked by target site duplications (5′-ACTRCCAR-3′or 5′-ACTRCCARTTA-3′), followed the CCTG repeat, suggesting that the repeat was originally derived from the *Alu* element insertion. In addition, our results revealed lineage-specific repetitive motifs: pyrimidine (CT)-rich repeat motifs in New World monkeys, dinucleotide (TG) repeat motifs in Old World monkeys and gibbons, and dinucleotide (TG) and tetranucleotide (TCTG and/or CCTG) repeat motifs in great apes and humans. Moreover, these di- and tetra-nucleotide repeat motifs arose from the poly (A) tail of the *Alu*Sx element, and evolved into unstable CCTG repeats during primate evolution. *Alu* elements are known to be the source of microsatellite repeats responsible for two other repeat expansion disorders: Friedreich ataxia and spinocerebellar ataxia type 10. Taken together, these findings raise questions as to the mechanism(s) by which *Alu*-mediated repeats developed into the large, extremely unstable expansions common to these three disorders.

## Introduction

Myotonic dystrophy type 2 (DM2) is an autosomal dominant multi-system disorder. It is caused by expansion of a tetranucleotide CCTG repeat in intron 1 of the zinc finger 9 (*ZNF9*) gene on chromosome 3q21 [1]. Patients with DM2 exhibit a wide range of phenotypes that include myotonia, muscle weakness, cardiac anomalies, cataracts, diabetes mellitus, and testicular failure [2]–[5]. In a normal allele, the repeat shows a complex motif with an overall configuration of (TG)*_n_*(TCTG)*_n_*(CCTG)*_n_*. The number of CCTG tracts is less than 30, with repeat interruptions of GCTG and/or TCTG motifs [6], and is stably transmitted from one generation to the next [1]. However, in the expanded allele, only the CCTG tract elongates, and the GCTG and TCTG interruptions disappear from the repeat tract. The sizes of expanded alleles are extremely variable, ranging from 75–11,000 repeats, with a mean of 5,000 repeats. The expanded DM2 alleles show marked somatic instability, with significant increases in length over time [1], [5]. Although the mechanism(s) underlying this unprecedented instability remains largely unknown, the uninterrupted CCTG repeat is prone to form a stable hairpin/dumbbell DNA structure and to expand due to an error in the recombination-repair mechanism [7]–[9]. To date, DM2 mutations have been identified predominantly in European Caucasians [6], [10]–[12]. Haplotype analysis indicates that the European DM2 mutations originate from a single founder, between approximately 4,000–11,000 years ago [10]. Liquori et al (2003) reported that humans, chimpanzees, gorillas, mice, and rats share a conserved DM2 repeat motif and flanking sequences, suggesting a conserved biological function [6]. However, the origin or evolutionary process of the DM2 repeat is still ambiguous.

A group of microsatellite repeat expansion disorders have been identified in the last two decades [13]. Most of these mutations involve unstable triplet repeats located in different regions of respective genes. The roles of repeat expansion mutations in the pathogenic mechanisms of these diseases are diverse and complex. Similar to DM2 [1], Friedreich ataxia (FRDA) and spinocerebellar ataxia type 10 (SCA10) are caused by large intronic expansions and show marked somatic and germ line instability [14]–[18]. Interestingly, in both FRDA and SCA10, *Alu* elements are proposed to be a source of the microsatellite repeats implicated in disease [19]–[23]. *Alu* elements are abundant in the human genome, with >1.1 million copies, and preferentially accumulate in gene-rich regions [24]. Due to this abundance, insertional mutation or unequal homologous recombination of *Alu* elements causes various inherited diseases [25].

To gain insight into the unstable DM2 repeat expansion mutation, we addressed the evolutionary history of the complex di- and tetra-nucleotide repeat configuration of (TG)*_n_*(TCTG)*_n_*(CCTG)*_n_* and the flanking *Alu* element.

## Results

**Figure 1:**
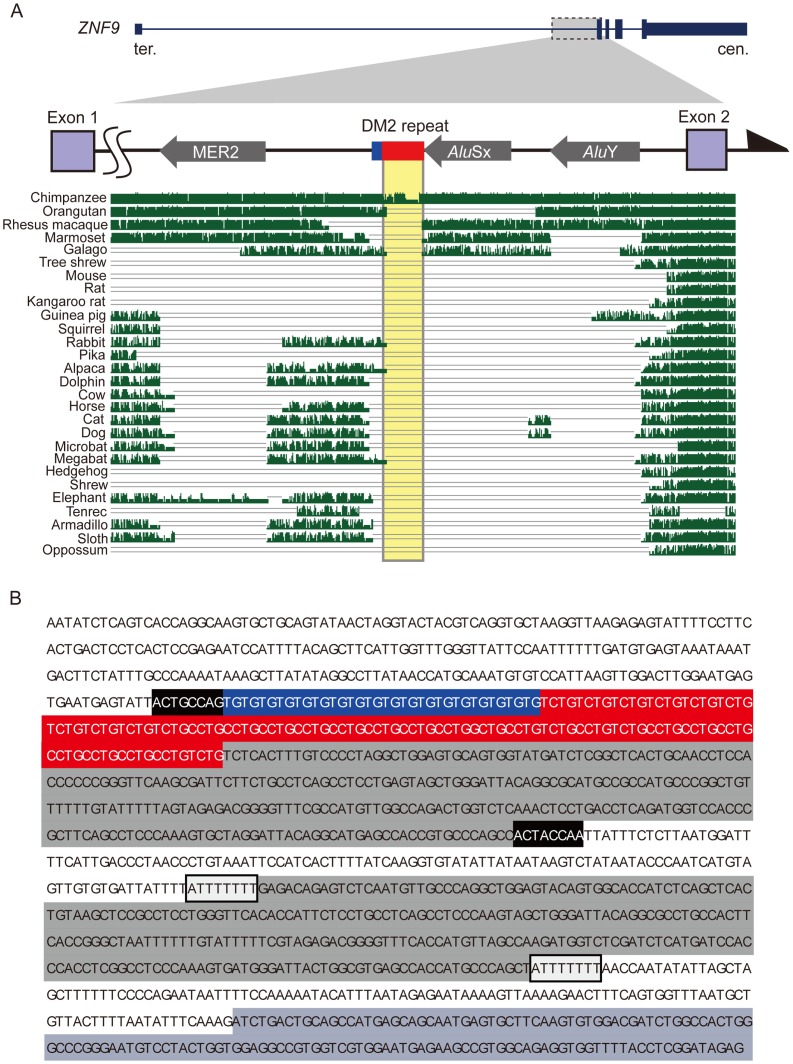
Genomic structure of the human *ZNF9* gene and repetitive elements in and around the DM2 (TG)*_n_*(TCTG)*_n_*(CCTG)*_n_* repeats. (A) Genomic alignment of the DM2 region from humans and other mammalian species. Filled thick arrows indicate DNA transposon (MER2) and short interspersed elements (*Alu*Sx and *Alu*Y). Blue, red, and purple boxes denote dinucleotide (TG), tetranucleotide (TCTG and CCTG) repeat regions, and the *ZNF9* exons, respectively. A yellow box highlights the regions corresponding to the human tetranucleotide repeat in other mammals. (B) Nucleotide sequence in and around the DM2 repeat of the human *ZNF9* gene. Each element is highlighted as follows: dinucleotide (TG) in blue; tetranucleotide (TCTG and CCTG) repeats in red; short interspersed elements (*Alu*Sx and *Alu*Y) in gray; and *ZNF9* exon 2 in purple. Black and white boxes flanking the *Alu*Sx and *Alu*Y elements, respectively, indicate target site duplications.

To define the mammalian origin of the DM2 (TG)*_n_*(TCTG)*_n_*(CCTG)*_n_* repeat (hereafter referred to as “DM2 repeat”), we compared human, chimpanzee, orangutan, rhesus macaque, marmoset, galago, tree shrew, mouse, rat, kangaroo rat, guinea pig, squirrel, rabbit, pika, alpaca, dolphin, cow, horse, cat, dog, microbat, megabat, hedgehog, shrew, elephant, tenrec, armadillo, sloth, and opossum genomes. We found repetitive elements of a DNA transposon and short interspersed repetitive elements (SINE), namely MER2, *Alu*Sx and *Alu*Y, located adjacent to the DM2 repeat, in inverse directions to the *ZNF9* reading frame (Figure 1A). The genomic region corresponding to the human TCTG and CCTG tetranucleotide repeats was entirely absent from other mammalian species except chimpanzees (yellow shaded box in Figure 1A). Interestingly, DM2 repeat was immediately adjacent to an *Alu*Sx element (Figure 1B), and target site duplications (TSDs) were observed at both ends of *Alu*Sx (5′-ACTRCCAR-3′; black-shaded box in Figure 1B) and *Alu*Y (5′-ATTTTTTT-3′; light gray-shaded box in Figure 1B). Because the DM2 repeat and *Alu*Sx are situated between the TSDs, the repeat itself is likely to have evolved from the *Alu*Sx and its poly (A) tail. It was reported that the DM2 repeat and the ∼200-bp 3′-flanking sequence are conserved among human, mouse, and rat [6]. While the 200-bp region is conserved between mouse and rat, we could not find the corresponding region in any other mammalian species (Figure S1A). Notably, the rodent dinucleotide (TG)_n_ repeat was followed by an identifier (ID) element (Figure S1B), which is a rodent-specific SINE [26], [27].

**Figure 2:**
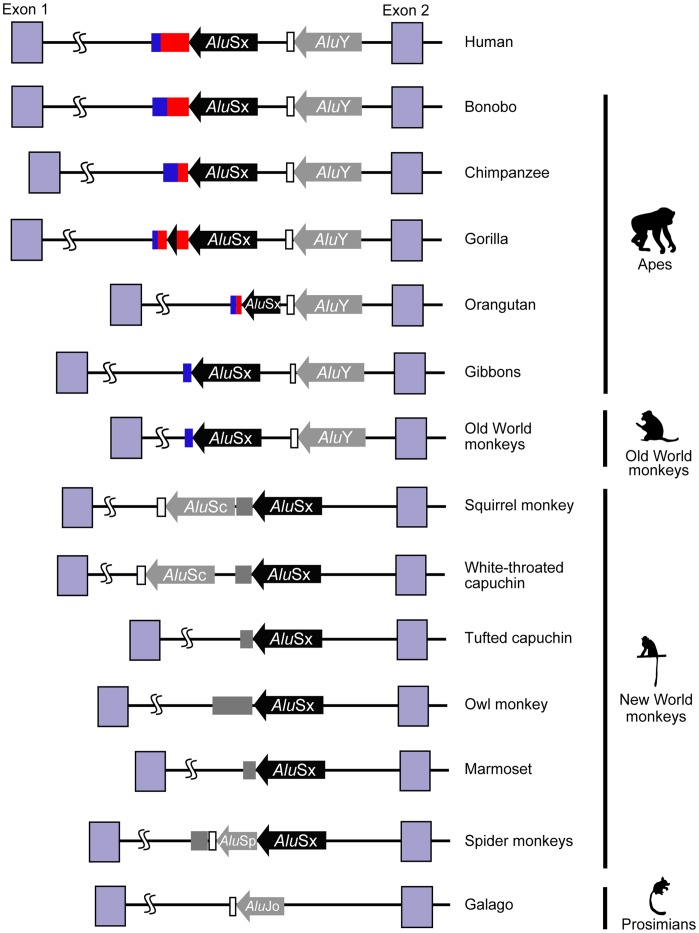
Diagram showing genomic structures of *Alu* insertions and the DM2-repeat region in intron 1 of the *ZNF9* gene in different primates. Black and gray arrows indicate *Alu*Sx and other *Alu* elements (*Alu*Y, *Alu*Sc, *Alu*Sp and *Alu*Jo), respectively. Blue, red, gray and white boxes denote dinucleotide (TG), tetranucleotide (TCTG and CCTG), pyrimidine (CT)-rich and poly (T) repeat regions, respectively.

**Figure 3:**
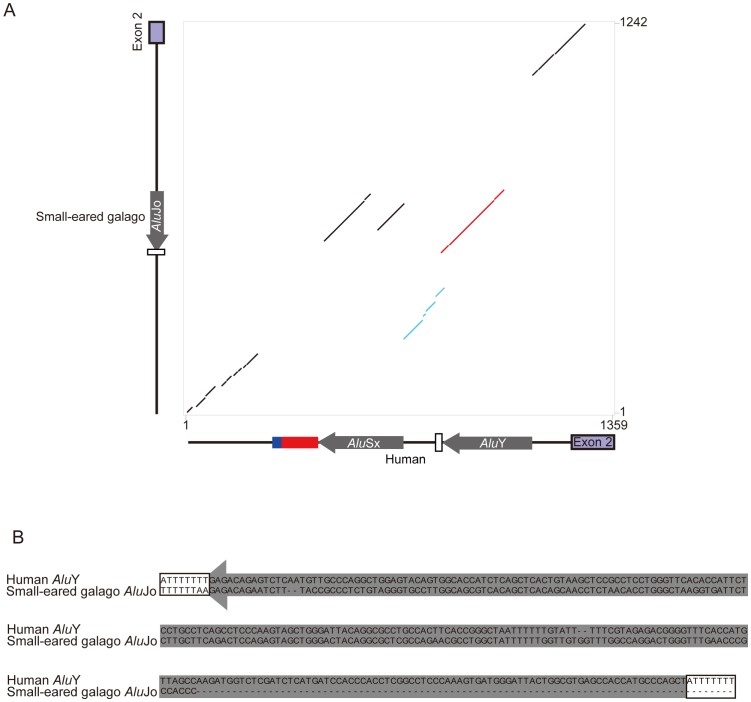
Nucleotide sequence comparison between human and prosimian intron 1 of the *ZNF9* gene. (A) Dot plot comparing the human *ZNF9* intron 1 (1359 bp, horizontal axis) with the corresponding region of the small-eared galago genome (1242 bp, vertical axis) by PipMaker. Blue, red, white boxes, and gray thick arrows denote dinucleotide (TG), tetranucleotide (TCTG and CCTG) repeat, poly (T) tract, and *Alu* element, respectively. Light blue dots indicate homologous region (56% identity) between the 131-bp region following human *Alu*Y and the 165-bp region following galago *Alu*Jo. Red dots indicates homologous region (67% identity) between human *Alu*Y and galago *Alu*Jo. (B) Sequence alignment between human *Alu*Y and small-eared galago *Alu*Jo. The aligned region corresponds to red dots shown in (A). *Alu* elements and flanking target site duplications are denoted as a gray thick arrow and white boxes, respectively. Dotted lines indicate sequence gaps.

To elucidate the origin of the DM2 repeat sequences in primate evolution, we next analyzed the sequence and genomic structure of *ZNF9* intron 1 in 26 primate species. PCR and sequence analysis revealed that DM2 repeats and the repeat surrounding regions varied considerably for examined primate species, except for Old World monkeys (Table 1, Figure 2, and Figure S2). Prosimian poly (T) tracts interrupted by AG and AA (5′-(T)_15_AG(T)_10_AA-3′), which seem to be the poly (A) tail of the *Alu* inserted into the opposite direction of the *ZNF9* gene, were followed by the *Alu* element. The poly (T) tracts were conserved in both the small-eared galago and the greater galago (Figure S3). RepeatMasker classified these *Alu* elements as *Alu*Jo, which is one of the oldest *Alu* elements [24]. The 165-bp region following the *Alu*Jo repeat in the small-eared galago is similar (56% identity) to the 131-bp region following human *Alu*Y (light blue dot plot in Figure 3A). Although the 5′-piece of prosimian *Alu*Jo was truncated (dotted line in Figure 3B), the 3′-piece of prosimian *Alu*Jo and TSD sequence were more similar to human *Alu*Y (56% identity) than those of *Alu*Sx (red dot plot in Figure 3A and Figure 3B), suggesting that prosimian *Alu*Jo (older *Alu* element) was inserted around the region where human *Alu*Y (younger *Alu* element) was inserted. On the other hand, there was no *Alu* element located on the region to corresponding to human *Alu*Y in New World monkeys (Figure 2 and dotted line in light gray-shaded boxes in Figure S4). Taken together, the prosimian *Alu*Jo was considered to have a different origin from *Alu*Sx and *Alu*Y and be retrotransposed independently into the same site of *Alu*Y inserted later in primate evolution.

**Table 1:** Sequence configurations of the DM2 repeat in 24 primates species.

Species	repeat configuration
Human	(TG)_18_(TCTG)_10_(CCTG)_8_GCTG(CCTG)TCTG(CCTG)TCTG(CCTG)_7_TCTG
**Apes**	
Chimpanzee	(TG)_23_(T)_2_(TG)_3_(T)_2_(TG)_4_TCTG(CCTG)_10_TCTG
Bonobo	(TG)_23_(TTTGCG)_2_TGTT(TG)_4_TC(TG)_3_(T)_2_(TG)_4_TCTG(CCTG)_3_(TCTG)_3_(CCTG)_11_(TCTG)_4_
Gorilla	(TG)_10_TCTG(TCTG)_3/4_TCTGCCTG(TCTG)_5_ (TC)_2_ ACTTTGTCCCCTAGGCTGGAGTGCAGTGGTATGA(TCTG)_5_(CCTG)_5/6_(TCTGCCTG)_2/1_(TCTG)_2_
Orangutan	(TG)_3_TATGTC(TG)_2_TC(TG)_2_TC(TCTG)_5_
Siamang	(T)_2_(TG)_4_TG/C(TG)_9_TA(TG)_2_(T)_2_(TG)_3_CCTGTCTG
Agile gibbon	(T)_2_(TG)_16/21_TA(TG)_2_(T)_2_(TG)_3_TCTG
White-handed gibbon	(T)_2_(TG)_10/13_TA(TG)_2_(T)_2_(TG)_4_TA(TG)_2_(T)_2_(TG)_3_TCTG
**Old World monkeys**	
Rhesus macaque	(TG)_3_CG(TG)_6_TATG
Bonnet macaque	(TG)_3_CG(TG)_6_TATG
De Brazza’s monkey	(TG)_3_CG(TG)_6_TATG
Patas monkey	(TG)_3_CG(TG)_8_TATG
Blue monkey	(TG)_3_CG(TG)_7_TATG
Hamadryas baboon	(TG)_3_CGTGAG(TG)_4_TATG
Mandrill	(TG)_3_CGTGAG(TG)_4_TATG
Silvered lutong	(TG)_3_CG(TG)_4_(TATG)_2_
Hanuman langur	(TG)_3_CG(TG)_4_(TATG)_2_
**New World monkeys**	
Common marmoset	C(T)_2_C(T)_4_C(T)_3_C(T)_2_C(T)_9_
Owl monkey	C(T)_2_(CCTT)_10_T(CTTT)_5_(CT)_5_(T)_2_(CTTTT)_2_(CTTT)_2_(CT)_7_(T)_4_(CT)_3_(T)_2_(CT)_2_(T)_4_(CT)_3_(T)_2_(CT)_2_(CTTT)_5_C(T)_6_
Squirrel monkey	C(T)_2_C(T)_4_(CTTT)_3_C(T)_4/5_
Tufted capuchin	C(T)_2_(CTTTT)_3_(T)_8/9_
White-throated capuchin	C(T)_2_(CTTTT)_3_(T)_3_(CTTTT)_3/4_(T)_7_
Black-handed spider monkey	C(T)_2_C(T)_4_(CTTT)_3/4_(T)_6/5_G(T)_5_
Long-haired spider monkey	C(T)_2_C(T)_4_(CTTT)_4_(T)_5/6_G(T)_5_

Contrary to prosimians, simians shared a common *Alu*Sx element (Figure 2) and its flanking TSDs consisting of 5′-ACTRCCAR-3′ or 5′-ACTRCCARTTA-3′ (black shaded box in Figure S4), although the 3′ TSDs was absent in Old World monkeys (dotted line in black shaded box in Figure S4). Although both *Alu*Sx and *Alu*Y were observed in Old World monkeys, apes, and human, *Alu*Y was completely absent in New World monkeys (Figure 2 and Figure S4), suggesting the *Alu*Y was inserted into the genome after the divergence of New World monkeys. Instead of the *Alu*Y insertion, additional *Alu*S insertions (*Alu*Sc insertions in white-throated capuchin and squirrel monkey, and *Alu*Sp insertions in black-handed and long-haired spider monkeys) were observed (Figure 2 and Figure S5). Since these additional *Alu*S insertions occurred in different sites and carried their own TSDs (blue, light green, and pink boxes in Figure S5), there were speculated to occur independently in each species of New World monkeys.

As with the DM2 repeat, a pyrimidine CT-rich sequence followed the 3′-end of *Alu*Sx in New World monkeys (Figure 2 and Table 1). In Old World monkeys and gibbons, the repeat motif consisted mainly of TG dinucleotides, while a single TCTG and/or CCTG sequence motif is present at the 3′-end of the repeat in gibbon sequences (Table 1). Orangutan, gorilla, bonobo and chimpanzee sequences also contain di- and tetra-nucleotide repeat motifs, similar to the human sequence. Of note, there was no CCTG motif in the orangutan sequence, and the TCTG motif did not constitute repetitive forms in the chimpanzee (Table 1). Interestingly, in the gorilla, 38 bp of the 3′-end of *Alu*Sx overlapped with the middle of the DM2 repeat (sequence underlined in Table 1), indicating that the duplication event occurred independently in the gorilla lineage.

## Discussion

*Alu* elements are primate-specific SINEs accounting for more than 10% of the human genome [28]. While most *Alu* elements lost their transpositional ability long ago, some active *Alu* elements can still increase their copy number. New insertions arise at a rate of approximately one in 20 births [29], [30]. Because there is no known mechanism specifically for *Alu* element excision, most remain in the genome as a record of ancient retrotransposition. Human *Alu* element is classified into subfamilies according to the insertion time from the oldest (*Alu*J) to intermediate (*Alu*S) and young (*Alu*Y) [24], [31]. A number of *Alu* elements are associated with microsatellite repeats; in fact, 5.7% of *Alu* poly (A) tails contain a patterned A-rich sequence such as (TA_3_)_n_, (CA_4_)_n_, (GA_3_)_n_, or (TA_2_)_n_ [32]. *Alu* elements are therefore suggested to be a source of microsatellite repeats [33], [34]. However, there are few examples indicating that the *Alu*-derived microsatellite repeat is responsible for human genetic disease.

In this study, we determined that *Alu*Sx and the associated complex DM2 repeat in the *ZNF9* gene are unique to primates, and are completely absent in other mammals (Figure 1A). This argues against previous findings that the complex repeat and the 3′-flanking region are conserved among humans, mice, and rats [6]. The corresponding region of the rodent dinucleotide (TG)_n_ repeat and the following 3′-flanking region are absent in other mammalian species (Figure S1A), and a rodent-specific ID element follows the dinucleotide repeat (Figure S1B). As a result, we conclude that the rodent dinucleotide repeat has a different origin from the primate DM2 repeat.

Among the primates, the *Alu*Sx element and the DM2 repeat are present in simians, humans, apes, Old World monkeys and New World monkeys (Figure 2). In addition, the *Alu*Sx and the complex repeat in the human *ZNF9* gene are flanked by TSDs (5′-ACTRCCAR-3′ or 5′-ACTRCCARTTA-3′; black-shaded boxes in Figure 1B and Figure S4). These findings indicate that the *Alu* element was retrotransposed into the genome very early in primate evolution, which coincides with the time that *Alu* elements explosively increased in number [24]. We also observed one of the oldest *Alu* elements, *Alu*Jo, in prosimian *ZNF9* intron 1 (Figure 2 and Figure 3). The small-eared galago and the greater galago have a similar pattern of *Alu*Jo insertion (Figure S3). The *Alu*Jo element in prosimians and the *Alu*Sx element in simians appear to have different origins, because the position of the *Alu*Jo and the 3′ flanking TSD are inconsistent with those of the *Alu*Sx, but rather more similar to those of the *Alu*Y (Figure 3). The time discrepancy between *Alu*Jo and *Alu*Y [24] also suggests that the *Alu*Jo element may have been independently retrotransposed into the prosimian lineage before divergence of the small-eared and greater galago.

**Figure 4:**
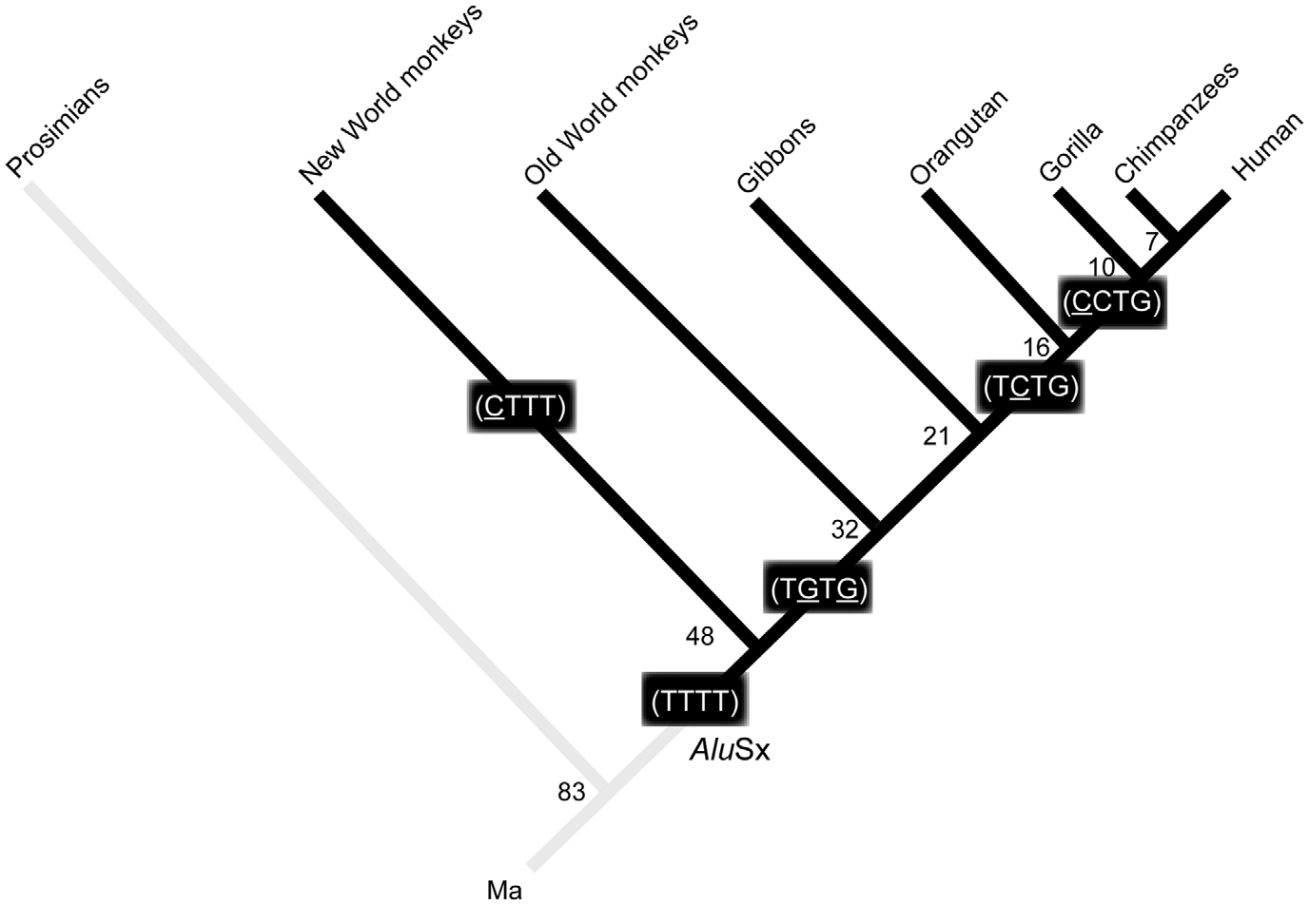
Evolutionary diagram of *ZNF9* repetitive motifs in the primate lineage. Evolutionary divergence after the *Alu*Sx retrotransposition event is indicated by dark bars. Parentheses imply multiple units. The number at each node represents divergence time according to TimeTree [44]. Time scale is in millions of years.

Focusing on the 3′-end of the simian *Alu*Sx element, we discovered pyrimidine (CT)-rich repetitive motifs in New World monkeys, (TG) dinucleotide repetitive motifs in Old World monkeys and gibbons, and (TG), (TCTG), and/or (CCTG) repetitive motifs in great apes and humans (Table 1). One of the most parsimonious scenarios of CCTG repeat evolution arises from these lineage-specific motifs (Figure 4). First, a poly (A) tail of *Alu*Sx was introduced into the genome in an inverse direction to the *ZNF9* gene, generating a TTTT repeat motif. Second, T to G substitution, or T to C and successive C to G substitution created a TGTG dinucleotide repeat motif in catarrhines. Next, G to C substitution created a TCTG repeat motif in great apes. Finally, sometime relatively recently, after the speciation of the *Pongo* genus, a C was introduced, resulting in a CCTG repeat motif. DM2 instability and associated pathogenicity likely occurred through the evolution of the repeat tract toward a stretch of tetranucleotide CCTG repeats in the primate genome, and the following loss of interruptions of TCTG and GCTG in the human mutant allele are thought to have acquired the DM2 instability and pathogenicity [1].

*Alu* dispersion throughout the genome provides opportunities for a higher level of unequal homologous recombination. *Alu*-mediated recombination is widely known as a source of local duplication and deletion [35], and is responsible for several human inherited disorders, including α-thalassaemia, Tay-Sachs disease and Duchenne muscular dystrophy [25]. In DM2, the mechanism of unequal crossing over has also been proposed to generate the long uninterrupted CCTG allele [7], [8], which is the basis of unstable expansion [36]. The primate-specific burst of *Alu* retrotransposition would initiate the expansion of segmental duplication in the gene-rich region, a possibility consistent with an *Alu*-to-*Alu* mediated recombination event. In fact, significant enrichment of *Alu* repeats is observed near or within the boundary of duplication sites in the human genome [37]. It is noteworthy that 38 bp of the 3′-end of the *Alu*Sx element showed duplication in the middle of the DM2 repeat in the gorilla sequence (Figure 2 and the underlined sequence in Table 1), implying that *Alu*Sx-mediated unequal crossing over occurred in the gorilla lineage.

Although *Alu* elements have been recognized as a source of various microsatellite repeats [32]–[34], there are, to date, only two known examples in which *Alu* elements underlie inherited microsatellite repeat expansion disorders: GAA triplet expansion in Friedreich ataxia (FRDA) derived from the middle A-rich site of *Alu* [19]–[22], and ATTCT pentanucleotide expansion in spinocerebellar ataxia type 10 (SCA10) from the poly (A) tail of *Alu* [17], [23]. Our results reveal that the DM2 CCTG tetranucleotide repeat is also derived from the 3′-end of the *Alu* element, similar to the ATTCT repeat. It is interesting that the repeats responsible for these three disorders commonly originate from the *Alu*Sx element [19], [20], [23]. Moreover, the *Alu*Sx insertion events occurred at approximately the same time for the three disorders, before the time of divergence of New World monkeys [20], [23]. This might be just a coincidence that all of the three are from *Alu*Sx, because *Alu*Sx (one of older *Alu* subfamilies [31]) are old enough to allow the time for evolutionary changes to create the types of repeats susceptible to expansion. Taken together, our data strengthen the evidence that *Alu* elements may be responsible for a wide variety of other hereditary microsatellite repeat expansion disorders, especially large non-coding repeat expansions [38], [39]. Because the characteristic common to DM2, FRDA and SCA10 is extremely unstable and large repeat expansions (up to thousands of repeats), the detailed molecular mechanism responsible for the instability of these *Alu*-mediated repeats warrants further investigation.

## Materials and Methods

### Ethics Statement

This study was carried out in accordance with the guideline for the use of non-human primate subjects, Primate Research Institute, Kyoto University. Blood samples were explicitly not taken for this study [23], [40]. The protocol was approved by the Ethical Committee of Nagoya University (#511).

### Samples

Non-human primate DNA samples were extracted by conventional phenol/chloroform methods from blood specimens from single individuals of five species of apes, eight species of Old World monkeys, six species of New World monkeys, and one prosimian species. These species were as follows: a bonobo (*Pan paniscus*), a gorilla (*Gorilla gorilla*), a siamang (*Symphalangus syndactylus*), a white-handed gibbon (*Hylobates lar*), an agile gibbon (*Hylobates agilis*), a patas monkey (*Erythrocebus patas*), a hamadryas baboon (*Papio hamadryas*), a mandrill (*Mandrillus sphinx*), a blue monkey (*Cercocebus mitis*), a bonnet macaque (*Macaca radiata*), a hunuman langur (*Semnopithecus entellus*), a de Brazza’s monkey (*Cercopithecus neglectus*), a silvered lutong (*Trachypithecus cristatus*), a white-throated capuchin (*Cebus capucinus*), a tufted capuchin (*Cebus apella*), an owl monkey (*Aotus trivirgatus*), a long-haired spider monkey (*Ateles belzebuth*), a black-handed spider monkey (*Ateles geoffroyi*), a squirrel monkey (*Saimiri sciureus*) and a greater galago (*Otolemur crassicaudatus*). All blood samples were obtained from Primate Research Institute, Kyoto University and extracted DNA samples were stored at The University of Tokyo Graduate School of Science until use.

### Genomic PCR and Sequencing of Primate *ZNF9* Genes

Genomic PCR reactions were performed in a 50 µl volume consisting of 1×buffer for KOD-plus- DNA polymerase, 15 pmol of genomic PCR primers (Table S1), 1 mM MgSO_4_, 200 µM of dNTP mixture, 5% dimethyl sulfoxide, 0.5 unit of KOD-plus- DNA polymerase (Toyobo, Osaka, Japan), and 10–100 ng of template DNA. The PCR conditions included an initial denaturing at 94°C for 2 min, followed by 35 cycles at 94°C for 30 sec, 54°C for 30 sec, and 68°C for 1 min 30 sec, with an additional extension at 68°C for 3 min. The amplified PCR fragments were gel purified using Wizard SV gel and PCR clean-up systems (Promega) and directly sequenced using a CEQ 8000 DNA sequence system (Bechman Caulter). For samples showing ambiguous sequences and heterozygosity, the PCR products were gel purified and cloned into a pTA2 plasmid vector using the TArget Clone-plus cloning system (Toyobo) and sequenced using sequencing primers (Table S1 and Table S2). Nucleotide sequences were deposited in the DDBJ/EMBL/GenBank (accession numbers AB595981–AB596009).

### Comparative *in silico* Analysis

The genomic alignments of mammalian *ZNF9* genes corresponding to the human *ZNF9* (TG)*_n_*(TCTG)*_n_*(CCTG)*_n_* repeat region or the mouse *Cnbp* (*Znf9*) dinucleotide repeat region were obtained from the University of California Santa Cruz (UCSC) Genome Browser (http://genome.ucsc.edu/). Besides the non-human primate species described above, sequence data from primates and rodents were also obtained from the Emsembl Genome Browser (http://www.ensembl.org/index.html): human (*Homo sapiens*; ENSG00000169714), chimpanzee (*Pan troglodyte*s; ENSPTRG00000015369), orangutan (*Pongo pygmaeus*; ENSPPYG00000013423), rhesus macaque (*Macaca mulatta*; ENSMMUG00000011585), common marmoset (*Callithrix jacchus;* ENSCJAG00000017430), small-eared galago (*Otolemur garnettii*; ENSOGAG00000005144), mouse (*Mus musculus*; ENSMUST00000032138), and rat (*Rattus norvegicus*; ENSRNOT00000013884). The sequences were aligned with ClustalX version 1.83 [41], and further edited manually using BioEdit version 7.0.5 [42] to verify the insertions and deletions. Dot plots were obtained using PipMaker, which is based on percent identity of each gap-free segment of sequence alignments generated by blastz [43]. Repetitive DNA sequences were classified using RepeatMasker (http://www.repeatmasker.org). The divergence times of each species were obtained by TimeTree [44].

## Supporting Information

Figure S1 **Genomic structure of the mouse** ***Cnbp*** **(** ***Znf9*** **) gene surrounding the dinucleotide (TG)_n_ repeat tract and the 200-bp 3′-flanking region in intron 1** [**6**] **with other species. (**A) Genomic alignment of the mouse *Cnbp* (*Znf9*) gene and the corresponding regions of other mammalian species. A Yellow box highlights the location of mouse dinucleotide (TG)n repeat and the 200-bp 3′-flanking region [6]. (B) Sequence alignment of the dinucleotide repeat and the 3′ flanking region in mouse and rat. A blue box and a gray thick arrow indicate the dinucleotide (TG)_n_ repeat and rodent-specific ID element, respectively.
(PDF)

Figure S2 **PCR analysis of intron 1 of the** ***ZNF9*** **gene of primates, including** ***Alu*** **elements and the DM2 region.** (A) Genomic structure spanning *ZNF9* exons 1 and 2. Arrows indicate PCR primers. (B) 1% Agarose gel electrophoresis of PCR-amplified genomic fragments from human, ape, Old World monkey, and New World monkey samples. “M” denotes 1 kb DNA ladder (Invitrogen).
(PDF)

Figure S3 **Sequence alignment of small-eared galago and greater galago.** A gray thick arrow and a white box indicate *Alu*Jo element and poly (T) tract, respectively.
(PDF)

Figure S4 **Multiple sequence alignment around the DM2 repeat region of human, apes, Old World monkeys, and New World monkeys.** A dark gray-shaded box, a light gray-shaded box, a purple box, black shaded boxes, and white boxes indicate *Alu*Sx, *Alu*Y, the *ZNF9* exon 2, target site duplications of *Alu*Sx, and target site duplications of *Alu*Y, respectively. A yellow box highlights the position of DM2 repeat sequences abbreviated as “REPEAT”. Dotted lines indicate sequence gaps.
(PDF)

Figure S5 **Multiple sequence alignment of seven species in New World monkeys showing species-specific** ***Alu*** **S insertions.** A yellow-shaded box, a purple-shaded box, a dark gray-shaded box, and light gray-shaded boxes indicate (CT)-rich repeat, the *ZNF9* exon 2, *Alu*Sx, and other *Alu*S insertions (*Alu*Sc insertions in white-throated capuchin and squirrel monkey, and *Alu*Sp insertions in black-handed and long-haired spider monkey), respectively. Target site duplications are shown on the both ends of *Alu* elements: *Alu*Sx in black-shaded boxes, *Alu*Sc of white-throated capuchin in blue boxes, *Alu*Sc of squirrel monkey in light green boxes, and *Alu*Sp of spider monkeys in pink boxes.
(PDF)

Table S1 **Primer sequences.**
(DOC)

Table S2 **Primers used for sequencing.**
(DOC)
