# Supplementary figures and images for: The Unstable CCTG Repeat Responsible for Myotonic Dystrophy Type 2 Originates from an AluSx Element Insertion into an Early Primate Genome
Source: PLoS One. 2012 Jun 19;7(6):e38379. doi: 10.1371/journal.pone.0038379 (PMC3378579; doi:10.1371/journal.pone.0038379)

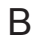

Mouse Rat

Mouse Rat

Mouse Rat

Supplement: Figure S1 — Genomic structure of the mouse Cnbp ( Znf9 ) gene surrounding the dinucleotide (TG)n repeat tract and the 200-bp 3′-flanking region in intron 1 [6] with other species. (A) Genomic alignment of the mouse Cnbp (Znf9) gene and the corresponding regions of other mammalian species. A Yellow box highlights the location of mouse dinucleotide (TG)n repeat and the 200-bp 3′-flanking region [6]. (B) Sequence alignment of the dinucleotide repeat and the 3′ flanking region in mouse and rat. A blue box and a gray thick arrow indicate the dinucleotide (TG)n repeat and rodent-specific ID element, respectively. (PDF) [file pone.0038379.s001.pdf]

A

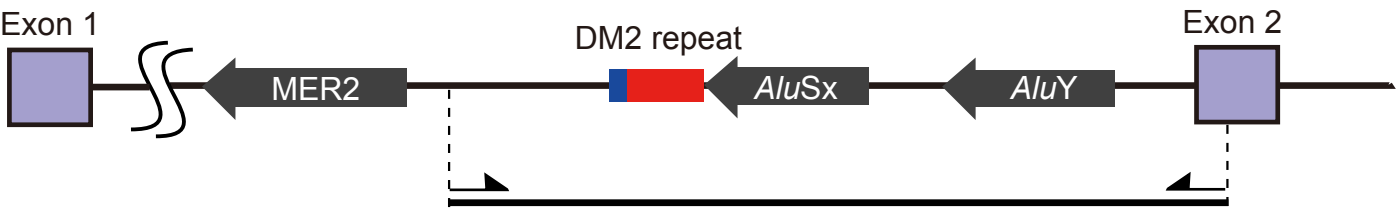

B

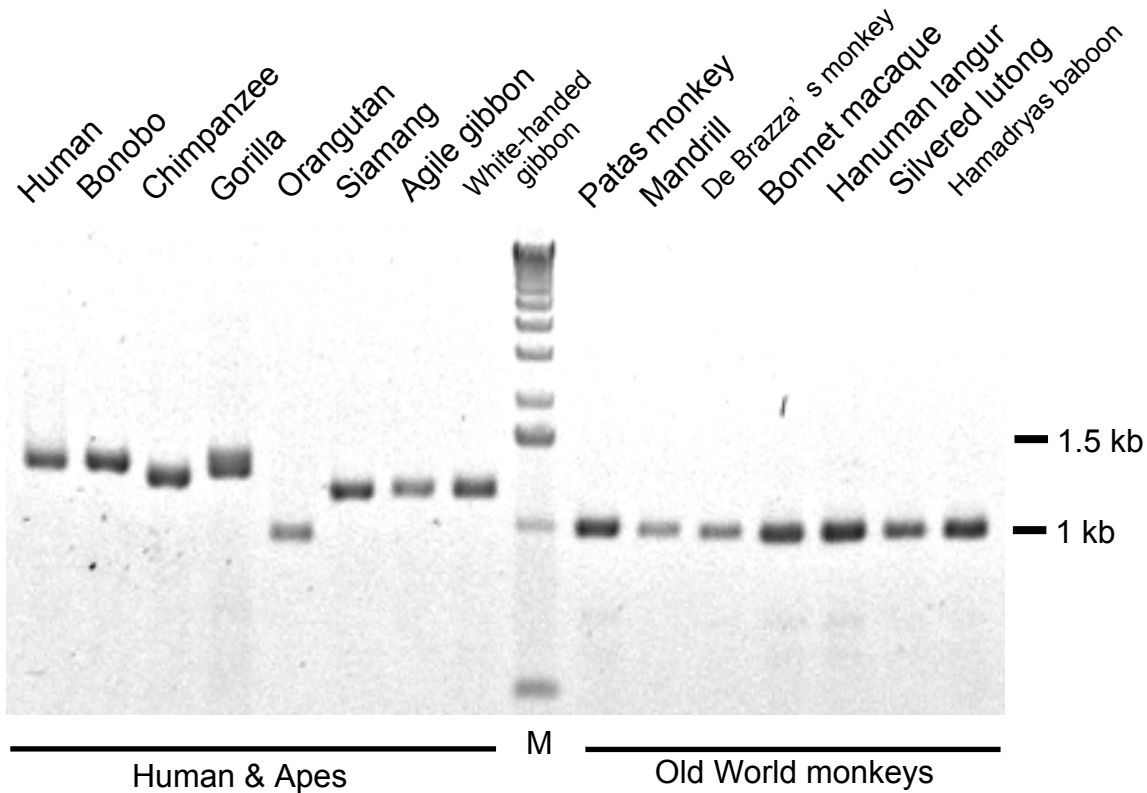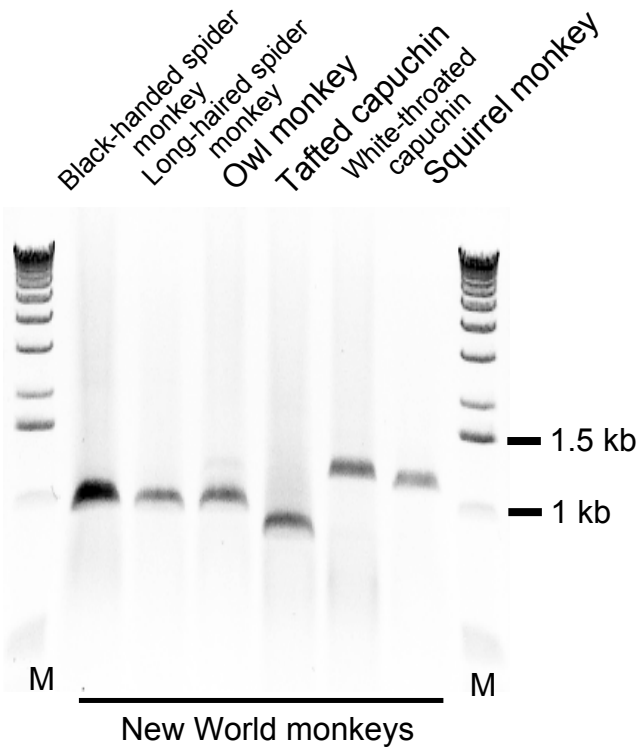

Supplement: Figure S2 — PCR analysis of intron 1 of the ZNF9 gene of primates, including Alu elements and the DM2 region. (A) Genomic structure spanning ZNF9 exons 1 and 2. Arrows indicate PCR primers. (B) 1% Agarose gel electrophoresis of PCR-amplified genomic fragments from human, ape, Old World monkey, and New World monkey samples. “M” denotes 1 kb DNA ladder (Invitrogen). (PDF) [file pone.0038379.s002.pdf]
