# Supplementary material for: The Unstable CCTG Repeat Responsible for Myotonic Dystrophy Type 2 Originates from an AluSx Element Insertion into an Early Primate Genome
Source: PLoS One. 2012 Jun 19;7(6):e38379. doi: 10.1371/journal.pone.0038379 (PMC3378579; doi:10.1371/journal.pone.0038379)
Supplement: Figure S3 — Sequence alignment of small-eared galago and greater galago. A gray thick arrow and a white box indicate AluJo element and poly (T) tract, respectively. (PDF) [file pone.0038379.s003.pdf]

Kurosaki et al. Figure S3

```
Small-eared galago TTTTTCTTTCACTGTTCTCTCACCCTCCATTGGTTTGGATTCATTCTGATTTTGTGATAAATAAATGATTTCATTTACTCAA
Greater galago     TTTTTCTTTCACTGTTCTCTCACCCTCCATTGGTTTGGATTCATTCTGATTTTGTGATAAATAAATGATTTCATTTACTCAA

Small-eared galago AGCTTATACAGTCTCATAACCATGGAAACATATCCGTTAAGTTGTACAACTTATGCTGTACAATCAGGTAT-CTCCAGGACC
Greater galago     AGCTTATACAGTCGCATAACCATGGAAACATATCCGTTAAGTTGTACAACTTATGCTGTACAATCAGGTATGCTCCAGGACC

Small-eared galago TGGAGATACTAATTACCAGTTATTTCTAATAATGGATGCTCATTGACACTAACCGAGTAAATCCTATCATTGTATGTGAGTC
Greater galago     TGGAGATACTAATTACCAGTTATTTCTAATAATGGATGCTCATTGACACTAACCGAGTAAATCTTATCATTGTATGTGAGTC

Small-eared galago ACTATGAAAGTTTGAGAC----------GTTCATTATTTCAATTCCAAGAAACCTAGTTGACAGCAACCTTTCTGATTGACC
Greater galago     ACTATGAAAGTTTGAGACTGTATTATTGGTTCATTATTTCAATTCCAAGAAACCTAGTTGACAGCAACCTTTCTGATTGACC

Small-eared galago TGTGCAAGTTTTTTTTTTTTTTTTTTTTTTTTGACCGGGGCTGGGTTTGAACCTGACACCTCCTGTATAGGGGGCCGGTGCC
Greater galago     TGTGCAAGTTTTTTTTTTTTTTTTTTTTTT--GACCGGGGCTGGGTTTGAACCTGACACCTCCTGTATAGGGGGCCGGTGCC

Small-eared galago CTAGTCACTGAGCTAGAGGCACCACCCTGTATCTTTTTTTTTTTTTTTAGTTTTTTTTTTAAGAGACAGAATCTTTACCGCC
Greater galago     CTAGTCACTGAGCTAGAGGCACCACCCTGTATCTTTTTTTTTTTTTTTAGTTTTTTTTTTAAGAGACAGAATCTTTACCGCC

Small-eared galago CTCTGTAGGGTGCCTTGGCAGCGTCACAGCTCACAGCAACCTCTAACACCTGGGCTAAGGTGATTCTCTTGCTTCAGACTCC
Greater galago     CTCTGTAGGGTGCCTTGGCAGCGTCACAGCTCACAGCAACCTCTAACACCTGGGCTAAGGTGATTCTCTTGCTTCAGACTCC

Small-eared galago AGAGTAGCTGGGACTACAGGCGCTCGCCAGAACGCCTGGCTATTTTTTGGTTGTGGTTTGGCCAGGACTGGGTTTGAACCCG
Greater galago     AGAGTAGCTGGGACTACAGGCGCTCGCCAGAACGCCTGGCTATTTTTTGGTTGTGGTTTGGCCAGGACTGGGTTTGAACCCG

Small-eared galago CCACCCTTGGTATATGGGGCTGGCACCCTACTCACTGAGCCACAGGCACTGCCCCGCCTGTGCTAGTTTTAACTTTTTCAGC
Greater galago     CCACCCTTGGTATATGGGGCTGGCACCCTACTCACTGAGCCACAGGCACTGCCCCGCCTGTGCTAGTTTTAACTTTTTCAGC

Small-eared galago TTATTGGTAGTGGTGGGAGGGTTTCATTTGTCCCTTCATGTAGATTATACATTTCTTGATTTTTGTGTATCTCAGAATTTTC
Greater galago     TTATTGGTAGTGGTGGGAGGGTTTCATTTGTCCCTTCATGTAGATTATACATTTCTTGATTTTTGTGTATCTCAGAATTTTC

Small-eared galago ATAATGACCCACACATTAGCATGTGTGTATTAATACATATATGCTTTATCTTACTGGTTTTAAAGATGAAAAACTGTAATGT
Greater galago     ATAATGACCCACACATTAGCATGTGTGTATTAATACATATATGCTTTATCTTACTGGTTTTAAAGATGAAAAACTGTAATGT

Small-eared galago ATATAATATATGATATATTATACATTATATATGTATATATAATATGCATTATGTAATTTTTCACTTTCAATATACTTTATAA
Greater galago     ATATAATATATGATATATTATACATTATATATGTATATATAATATGCATTATGTAATTTTTCACTTTCAATATACTTTATAA

Small-eared galago TAGGTCTGTTATGCCTAAGTATATAATTGTGCAGTTGTAAAAAATAATATATTGGCCAGTTTTTCCCTAGAATAATTTTCC
Greater galago     TAGGTCTGTTATGCCTAAGTATATAATTGTGCAGTTGTAAAAAATAATATATTGGCCAGTTTTTCCCTAGAATAATTTTCC

Small-eared galago AAAAATACTTTTAAAGGGGAATCAAAGAAGTAATTTTCAGTGGCTTAATGTTGTTATTTTTCATTTTTTAAAG Exon 2
Greater galago     AAAAATACTTTTAAAGGGGAATCAAAGAAGTAATTTTCAGTGGCTTAATGTTGTTATTTTTCATTTTTTAAAG
```
