# Supplementary material for: The Unstable CCTG Repeat Responsible for Myotonic Dystrophy Type 2 Originates from an AluSx Element Insertion into an Early Primate Genome
Source: PLoS One. 2012 Jun 19;7(6):e38379. doi: 10.1371/journal.pone.0038379 (PMC3378579; doi:10.1371/journal.pone.0038379)

Kurosaki et al. Figure S4

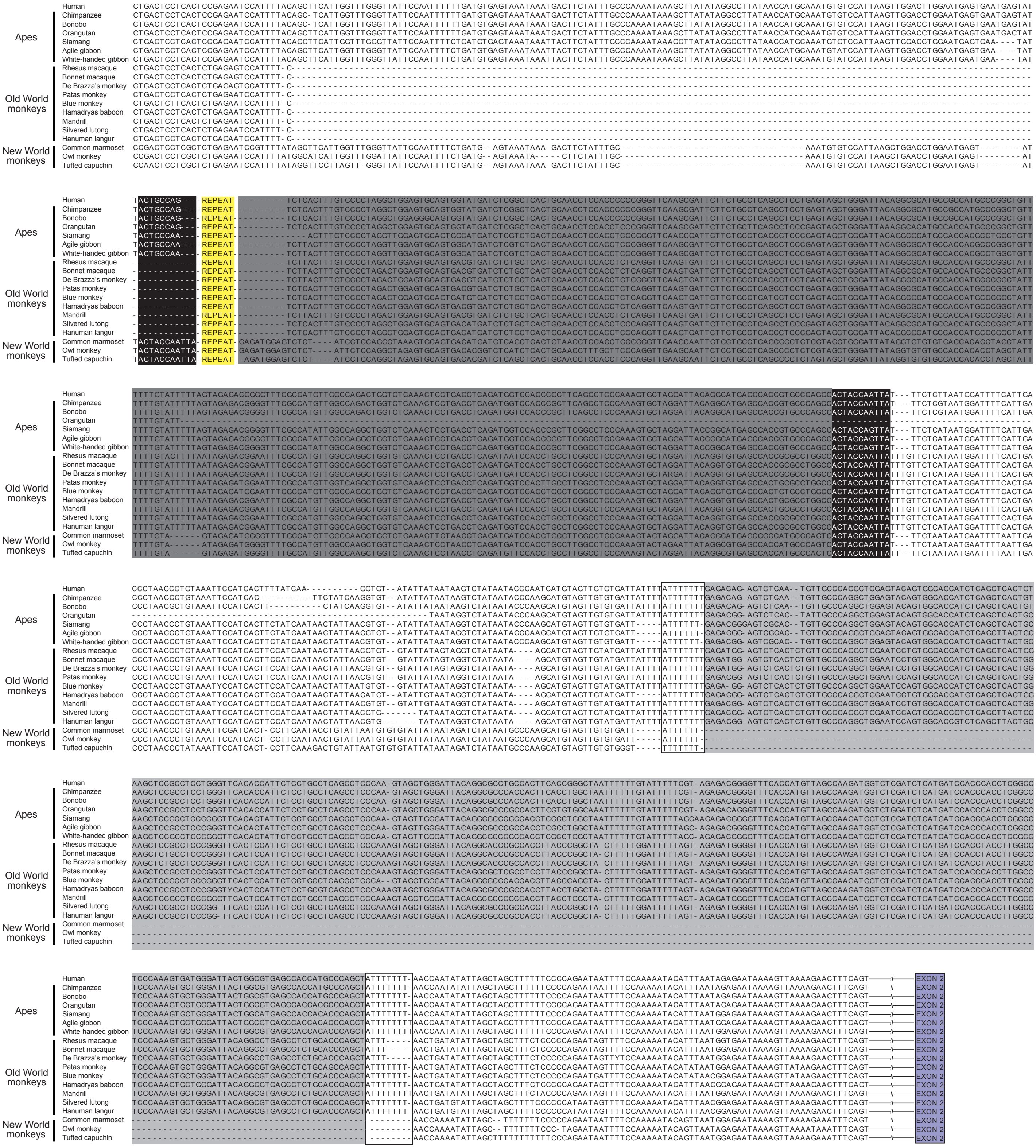

Supplement: Figure S4 — Multiple sequence alignment around the DM2 repeat region of human, apes, Old World monkeys, and New World monkeys. A dark gray-shaded box, a light gray-shaded box, a purple box, black shaded boxes, and white boxes indicate AluSx, AluY, the ZNF9 exon 2, target site duplications of AluSx, and target site duplications of AluY, respectively. A yellow box highlights the position of DM2 repeat sequences abbreviated as “REPEAT”. Dotted lines indicate sequence gaps. (PDF) [file pone.0038379.s004.pdf]
