# Supplementary material for: The Unstable CCTG Repeat Responsible for Myotonic Dystrophy Type 2 Originates from an AluSx Element Insertion into an Early Primate Genome
Source: PLoS One. 2012 Jun 19;7(6):e38379. doi: 10.1371/journal.pone.0038379 (PMC3378579; doi:10.1371/journal.pone.0038379)
Supplement: Table S1 — Primer sequences. (DOC) [file pone.0038379.s006.doc]

|  | **Primer sequences** |
| --- | --- |
| Genomic PCR primers for simians | F1: 5′-GCCAGATCGTCCACACTTGAA-3′ |
|  | R1: 5′-AATATCTCAGTCACCAGGCAAG-3′ |
| Genomic PCR primers for prosimians | F1: 5′-GCCAGATCGTCCACACTTGAA-3′ |
|  | R2 : 5′-CTATATCTCAGTCATGAGGCAGG-3′ |
| Sequencing primers | F2: 5′-TCATTGCTGCTCATGGCTGC-3′ |
|  | F3: 5′-CATTAAACCACTGAAAGTTCTT-3′ |
|  | F4: 5′-GTTGCAGTGAGCCGAGATCA-3′, |
|  | F5: 5′-GCCTDTAATCCCAGCTACTC-3′ |
|  | F6: 5′-ACCTATAATCCCAGCTACKA-3′ |
|  | F7: 5’-AGGTGTTCAGGTTGCAGGAG-3’ |
|  | F8: 5’-CTATATCTCAGTCATGAGGCAGG-3’ |
|  | F9: 5’-TTCTTTCACTGTTCTCTCAC-3’ |
|  | F10: 5’-CCTAGTTGACAGCAACCTTTCTG-3’ |
|  | R3: 5′-TCAGGTGCTAAGGTTAAGAG-3′ |
|  | R4: 5′-TGATCTCGGCTCACTGCAAC-3′ |
|  | R5: 5′-TAGCTGGGRTTATAGGTGTG-3′ |
|  | R6: 5’-CATTAAGCCACTGAAAATTACT-3’ |
|  | R7: 5’-CACACATGCTAATGTGTGGGTC-3’ |
|  | T7: 5’-TAATACGACTCACTATAGGG-3’ |
|  | M13R: 5’-CAGGAAACAGCTATGAC-3’ |

Table S1. Primer sequences.
