# Supplementary material for: The Unstable CCTG Repeat Responsible for Myotonic Dystrophy Type 2 Originates from an AluSx Element Insertion into an Early Primate Genome
Source: PLoS One. 2012 Jun 19;7(6):e38379. doi: 10.1371/journal.pone.0038379 (PMC3378579; doi:10.1371/journal.pone.0038379)
Supplement: Table S2 — Primers used for sequencing. (DOC) [file pone.0038379.s007.doc]

Table S2. Primers used for sequencing.

| **Species** | **Primers used for sequencing** |
| --- | --- |
| **Apes** |  |
| Bonobo | F1, F2, F5, R1, R3, R4, M13R |
| Gorilla | F1, F2, F3, F5, R1, R3, R4, M13R |
| Siamang | F1, F2, F5, R1, R3, R4, M13R |
| Agile gibbon | F1, F2, F5, R1, R3, R4, M13R |
| White-handed gibbon | F1, F2, F5, R1, R3, R4, M13R |
| **Old World monkeys** |  |
| Bonnet macaque | F1, F4, F5, R1, R3, R4 |
| De Brazza’s monkey | F1, F2, F4, R1, R3, R4 |
| Patas monkey | F1, F2, F5, R1, R3, R4 |
| Blue monkey | F1, F2, F5, R1, R3, R4 |
| Hamadryas baboon | F1, F2, F5, R1, R3, R4 |
| Mandrill | F1, F2, F5, R1, R3, R4 |
| Silvered lutong | F1, F2, F5, R1, R3, R4 |
| Hanuman langur | F1, F2, F3, F5, R1, R3, R4 |
| **New World monkeys** |  |
| Owl monkey | F1, F6, R1, R3, R4, R5 |
| Squirrel monkey | F1, F3, F6, R1, R3, R5 |
| Tufted capuchin | F1, F6, R1, R4, R5 |
| White-throated capuchin | F1, F3, F6, R1, R4, R5 |
| Black-handed spider monkey | F1, F3, F6, R1, R4, R5 |
| Long-haired spider monkey | F1, F2, F3, F6, R1, R3, R4, R5, T7 |
| **Prosimians** |  |
| Greater Galago | F1, F2, F7, F8, F10, R2, R6, R7, T7 |
