# Supplementary material for: The Unstable CCTG Repeat Responsible for Myotonic Dystrophy Type 2 Originates from an AluSx Element Insertion into an Early Primate Genome
Source: PLoS One. 2012 Jun 19;7(6):e38379. doi: 10.1371/journal.pone.0038379 (PMC3378579; doi:10.1371/journal.pone.0038379)

Kurosaki et al. Figure S5

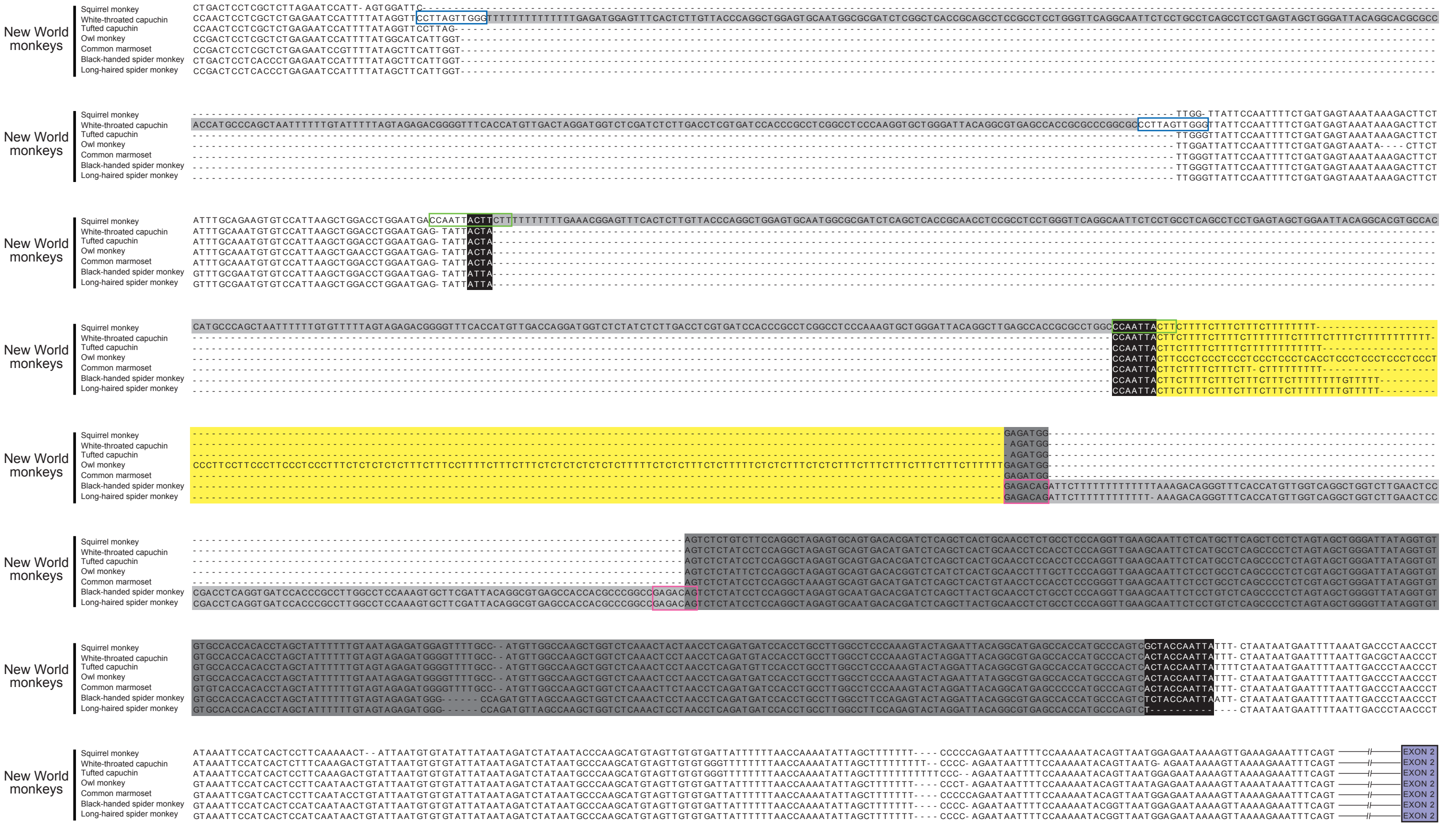

Supplement: Figure S5 — Multiple sequence alignment of seven species in New World monkeys showing species-specific Alu S insertions. A yellow-shaded box, a purple-shaded box, a dark gray-shaded box, and light gray-shaded boxes indicate (CT)-rich repeat, the ZNF9 exon 2, AluSx, and other AluS insertions (AluSc insertions in white-throated capuchin and squirrel monkey, and AluSp insertions in black-handed and long-haired spider monkey), respectively. Target site duplications are shown on the both ends of Alu elements: AluSx in black-shaded boxes, AluSc of white-throated capuchin in blue boxes, AluSc of squirrel monkey in light green boxes, and AluSp of spider monkeys in pink boxes. (PDF) [file pone.0038379.s005.pdf]
